# Strengths, Weaknesses, Opportunities, and Threats (SWOT) of Implementing Teleconsultation: A Systematic Review

**DOI:** 10.1002/hsr2.70645

**Published:** 2025-05-05

**Authors:** Shiva Khoshsirat, Hassan Soleimanpour, Peyman Rezaei‐Hachesu

**Affiliations:** ^1^ Department of Health Information Technology School of Management and Medical Informatics, Tabriz University of Medical Sciences Tabriz Iran; ^2^ Medical Philosophy and History Research Center, Emergency and Trauma Care Research Center Imam Reza General Hospital, Tabriz University of Medical Sciences Tabriz Iran

**Keywords:** COVID‐19, opportunity, strength, teleconsultation, threat, weakness

## Abstract

**Background and Aims:**

The COVID‐19 pandemic has changed the traditional models of providing services in health systems. One of the recommended ways to provide healthcare services in this era is teleconsultation. This study aimed to determine the strengths, weaknesses, opportunities, and threats (SWOTs) of teleconsultation from the general practitioner's point of view and to implement it in the COVID‐19 era.

**Methods:**

A systematic review was conducted by searching online databases, including the PubMed, Scopus, and WOS databases, from the beginning to January 1, 2024, without restrictions and using relevant keywords. All studies that mentioned at least one of the areas of strength, weakness, opportunities, or threats related to teleconsultation were included in the study. We used content analysis to combine the results.

**Results:**

Ultimately, 32 studies were included in this review. The most important factors were determined in four domains. Strengths included ease of use of technology, reduction of time and cost, and facilitating documentation. Weaknesses included a lack of physical exams, less direct communication, and internet‐related problems. The opportunities included the increasing progress of medical technologies worldwide, continuity of care, and people's interest in the daily use of new technologies. Threats included sociocultural barriers, the need for continuous training, and competing interests.

**Conclusion:**

Examining internal and external factors is important for formulating a plan. Identifying these factors and using them can lead to the formulation of effective and efficient programs in the field of teleconsultation for general practitioners in the era of COVID‐19. Without paying attention to these issues, adopting appropriate plans to minimize weaknesses and threats, and effectively using strengths and opportunities to implement telemedicine projects, there is a possibility of failure and waste of time, effort, and credit in the health sector.

## 1 Background

Telemedicine or telehealth is defined as providing medical and health services remotely via technology. Teleconsultation is a form of telemedicine in which digital information and remote technologies are used to deliver information between service deliverers and caregivers. In a teleconsultation system, patients can have consultations with all types of service providers, including physicians, nurses, and allied health providers, from their homes or places that are private [1, 2, 3, 4].

Telemedicine offers the possibility of consulting specialists for diagnosis, discussion, and exchange of opinions on the diagnosis of diseases and review of treatment protocols for patients. Telemedicine can lead to a reduction in rework in performing tests and photos such as radiology, saving patient and insurer costs, allowing more access to information related to the health of patients, and providing treatment options and preventive policies for getting sick. Access to healthcare and reduce health inequalities, reduce referrals and save time for patients [5, 6, 7].

Working with remote systems has problems that vary depending on the type of system and the service provided. Additionally, these systems have different strengths and weaknesses. The most important weaknesses related to these systems are related to the existing infrastructure related to the Internet, which varies depending on the speed of the Internet. In addition, the absence of a patient and lack of physical examination are considered important weaknesses. The most important strengths of using these remote systems include reducing costs and not needing to visit people, which were highly important during the COVID‐19 pandemic because it is recommended that social distancing be maintained as one of the most effective methods of prevention [8, 9, 10, 11, 12, 13, 14].

The spread of the virus and efforts to reduce its spread have changed the way health services operate around the world, and many of these changes are likely to persist long after the crisis subsides [15]. One of those changes is the transition from traditional consultation to teleconsultation [16, 17, 18]. The success of telemedicine services is subject to a variety of factors, from technical issues such as infrastructure, legislation, change management, and business models [19]. Proper diagnosis and management of this disease can be an important intervention in reducing the mortality of critically ill patients with COVID‐19 [20].

Considering the expansion of the use of these remote systems in the field of medicine and for the prevention and treatment of patients in the era of COVID‐19, understanding the problems related to these systems and identifying the existing internal and external factors can aid in the optimal use of these systems. Therefore, this study aimed to determine the strengths, weaknesses, opportunities, and threats (SWOT) of teleconsultation from the general practitioner's (GPs) point of view and to implement it in the COVID‐19 era.

## 2 Materials and Methods

We conducted this systematic review following the Preferred Reporting Items for Systematic Reviews and Meta‐Analyses (PRISMA) guidelines [21].

### 2.1 Eligibility Criteria

All original studies that reported at least one of the strengths, weaknesses, opportunities, or threats of teleconsultation and inspected its feasibility were included in this study. Only studies whose full text was accessible were reviewed. Studies whose languages were other than English or Farsi were excluded.

### 2.2 Information Sources and Search

Three main databases, PubMed, Scopus, and WOS, searched from inception to January 1, 2024. We searched databases including SID and Magiran to identify relevant articles in Farsi. Additionally, we searched the references of the included studies and contacted the authors of the articles for which the full text was unavailable. Search strategies, including telemedicine, telehealth, teleconsultation, electronic consultation, remote consultation, eHealth, eMedicine, strengths, weakness, opportunity, treat, and SWOT analysis, were combined using Boolean operators of OR, AND, and NOT. The full search strategy for the PubMed database is available in Table 1.

**Table 1:** The results of the search strategy for the database.

Database	Query	Records
PubMed	((“Telemedicine”[Title/Abstract] OR “Telehealth”[Title/Abstract] OR “Teleconsultation”[Title/Abstract] OR “electronic consultation”[Title/Abstract] OR “remote consultation”[Title/Abstract] OR “eHealth”[Title/Abstract] OR “eMedicine”[Title/Abstract]) AND (“strengths”[Title/Abstract] OR “weaknesses”[Title/Abstract] OR “Opportunities”[Title/Abstract] OR “Threats”[Title/Abstract] OR “SWOT”[Title/Abstract])) AND (1993:2022[pdat])	2283

### 2.3 Selection Process

All the records were imported to EndNote version X9, after which duplicates were removed. Then, all the articles were reviewed based on the title, abstract, and full text, taking into account the inclusion and exclusion criteria. All procedures were performed by the two reviewers independently. To check for differences between the two reviewers, the opinions of the third reviewer were used.

### 2.4 Data Extraction

The following items were extracted using two independent reviewers: first author, publication date, study design, country of origin, study area, and major findings related to the SWOT of Teleconsultation.

### 2.5 Synthesis Methods

Considering that the data collected in this study were qualitative, we used the qualitative content analysis (QCA) method to analyze the results. First, items related to strengths, weaknesses, threats, and opportunities were extracted from all individual studies and then combined using the content analysis method. We conducted QCA following Mayring's (2014) inductive‐deductive protocol for systematic qualitative synthesis, which is widely used in health services research. The process adhered to the six‐step framework: (1) defining the research problem as identifying and categorizing SWOT factors of teleconsultation from GPs perspectives, with text segments (e.g., sentences or paragraphs describing SWOT factors) serving as the units of analysis; (2) formulating guiding questions (e.g., “What factors influence teleconsultation adoption?”); (3) determining variables by mapping emergent themes to SWOT domains (e.g., “cost reduction” as a strength); (4) coding, where two reviewers independently extracted and coded text segments using NVivo 12 software, first inductively generating codes (e.g., “lack of physical examination”) and then deductively categorizing them into SWOT domains; (5) categorization, grouping codes into subthemes (e.g., “technical barriers” encompassing “internet instability” and “software usability”) and calculating inter‐coder reliability (Cohen's *κ* = 0.81); and (6) reporting validated themes. Coding discrepancies (12% of cases) were resolved through consensus discussions with a third reviewer. To ensure rigor, we followed the Standards for Reporting Qualitative Research (SRQR) [22], maintained a reflexive codebook, and documented audit trails for transparency and reproducibility.

### 2.6 Study Selection

**Figure 1:**
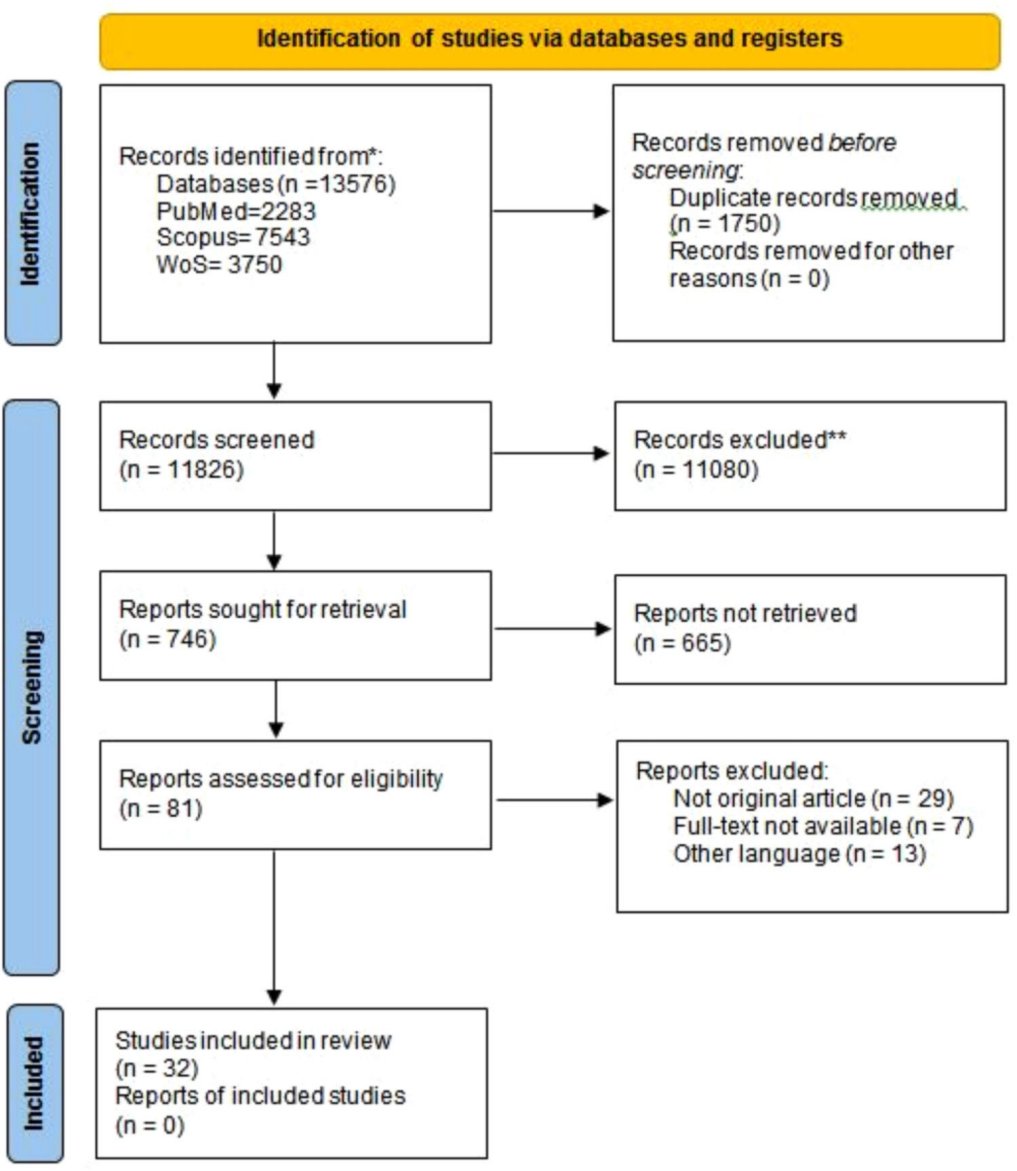
PRISMA flow diagram.

After all the considered databases were searched, 13,576 records were found. Of these, 1750 were duplicated and removed. A total of 11,826 records were screened, and 746 of those records were sought for retrieval. After retrieval, 121 full texts were assessed based on the eligibility criteria. Finally, 32 articles were included in this study [23, 24, 25, 26, 27, 28, 29, 30, 31, 32, 33, 34, 35, 36, 37, 38, 39, 40, 41, 42, 43, 44, 45, 46, 47, 48, 49, 50, 51, 52, 53, 54]. The PRISMA flow diagram of the selection process for the systematic review is shown in Figure 1.

## 3 Results

A total of 32 articles from 21 countries were included in this study. The largest number of articles were related to the US (12.5%), Brazil (12.5%), and Germany (12.5%). The largest number of articles were published in 2022 (37.5%), 2021 (21.8%), and 2020 (9.3%). In terms of design, most of the studies were qualitative (31.1%), followed by cross‐sectional (21.8%). Funding and ethical statements were reported in 71.8% and 65.6, respectively, of the included studies. Table 2 shows the summary characteristics of the included studies.

**Table 2:** Summary characteristics of the included studies.

First author/year	Country	Design	Data collection	Sample size	Title	Journal
Alboraie et al. (2021)	Egypt	Cross‐sectional	May to July 2020	686	Telemedicine knowledge, application, and barriers in Egypt: A national survey. International Journal of Telemedicine and Applications	*International Journal of Telemedicine and Applications*
Anderson and Singh (2021)	US	Case study	NR^a^	NR	A case study of using telehealth in a rural healthcare facility to expand services and protect the health and safety of patients and staff	*Healthcare*
Back et al. (2022)	Germany	Pilot project	2018 to 2020	NR	Implementation of online video consultations in a regional health network: A management feasibility analysis from an orthopedic perspective	*BMC Health Services Research*
Caetano et al. (2020)	Brazil	Multimethods	2020	NR	Challenges and opportunities for telehealth during the COVID‐19 pandemic: Ideas on spaces and initiatives in the Brazilian context	*Reports in Public Health*
Combi et al. (2016)	Italy	Survey	NR	NR	Telemedicine for developing countries	*Applied Clinical Informatics*
Comfort et al. (2022)	US	Survey	April 2020 to January 2021	907	Assessing differences in contraceptive provision through telemedicine among reproductive health providers during the COVID‐19 pandemic in the United States	*Reproductive Health*
Correia et al. (2017)	Cape Verde	Qualitative study	2013−2014	24	[Implementation of telemedicine in Cape Verde: Influencing factors]	*Acta Medica Portuguesa*
de Melo et al. (2018)	Brazil	Qualitative study	NR	30	Belo Horizonte telehealth: Incorporation of teleconsultations in a health primary care system	*Telemedicine and e‐Health*
Duking et al. (2018)	Germany	Qualitative study	NR	NR	The potential usefulness of virtual reality systems for athletes: A short SWOT analysis	*Frontiers in Physiology*
Fatima et al. (2022)	Pakistan	Cross‐sectional	August 2020 to January 2021	114	COVID‐19 pandemic and the use of tele‐health by pediatricians and pediatric post graduate residents	*Journal of University Medical & Dental College*
Filfilan et al. (2021)	France	Prospective study	May 2020	80	Positive environmental impact of remote teleconsultation in urology during the COVID‐19 pandemic in a highly populated area	*Progrès en Urologie*
Ghilencea et al. (2022)	Italy	Qualitative study	2020−2021	NR	Telemedicine: Benefits for cardiovascular patients in the COVID‐19 era	*Frontiers in Cardiovascular Medicine*
Gray et al. (2016)	Canada, Scotland, and the US	Case study	NR	NR	eHealth advances in support of people with complex care needs: Case examples from Canada, Scotland, and the US	*Healthcare Quarterly*
Kazi et al. (2020)	Pakistan	Mixed‐method	NR	51	Current challenges of digital health interventions in Pakistan: Mixed methods analysis	*Journal Of Medical Internet Research*
Hussein and Kalifa (2012)	Egypt	Qualitative study	NR	NR	Telemedicine in Egypt: SWOT analysis and future trends. GMS Med Inform Biom Epidemiol	*GMS Medizinische Informatik, Biometrie und Epidemiologie*
Kim et al. (2021)	Korea	Survey	February 2020 to June 2020	567,390	Preliminary results of teleconsultations temporarily allowed during the COVID‐19 pandemic	*Yonsei Medical Journal*
Koch et al. (2022)	Brazil	Medical record review	June 2020 and March 2021	161	Teleconsultation at a public ophthalmic teaching hospital during the COVID‐19 pandemic	*Arquivos Brasileiros de Oftalmologia*
Mihova (2011)	Bulgaria	Qualitative study	NR	23	Telemedical solutions‐practical approach in Bulgaria	*Intech Open*
Milcent and Zbiri (2022)	France	Survey	March 2020 to April 2020	247	Use of telehealth: Evidence from French teleconsultation for women's healthcare, prior to and during COVID‐19 pandemic	*Health Services Management Research*
Negi et al. (2020)	India	Cross‐sectional	April to July 2020	131	Perception of patients getting teleconsultation in an e‐OPD during COVID pandemic. Indian J Pharm Pharmacol	*Indian Journal of Pharmacy and Pharmacology*
Ngassa Piotie et al. (2022)	South Africa	Qualitative study	April to June 2021	31	Factors affecting the implementation of a complex health intervention to improve insulin management in primary care: A SWOT analysis	*African Journal of Primary Health Care & Family Medicine*
Peixoto et al. (2021)	Brazil	Cross‐sectional	September 2020	527	Drivers for teleconsultation acceptance in Brazil: Patients’ perspective during the COVID‐19 pandemic	*Revista de Administração Contemporânea*
Perwitasari and Susanti (2019)	Indonesia	Qualitative study	NR	NR	The role of space technology to telemedicine in Indonesia toward the goal of sustainable development	*International Journal of Innovative Science and Research Technology*
Qi Tan et al. (2022)	Singapore	Qualitative study	July to November 2021	22	Enablers and barriers to nurse‐facilitated geriatric teleconsultations in nursing homes: A qualitative descriptive multisite study	*Age and Aging*
Reinecke et al. (2021)	Germany	Survey	August 2018 and December 2019	1055	Acceptance, barriers, and future preferences of mobile health among patients receiving trauma and orthopedic surgical care: Paper‐based survey in a prospective multicenter study	*JMIR Mhealth and Uhealth*
Särchen et al. (2022)	Germany	Qualitative study	June 2021	12	Patient care via video consultations: Piloting and SWOT analysis of a family medicine digitally synchronous seminar for medical students	*International Journal of Environmental Research and Public Health*
Saxena et al. (2022)	India	Cross‐sectional	April 2020 to October 2020	5278	Strength, weakness, opportunities, and threats (SWOT) analysis of virtual outpatient department under telemedicine department during the COVID‐19 pandemic	*Cureus*
Sayani et al. (2019)	Afghanistan, Pakistan, Tajikistan, and the Kyrgyz Republic	Cross‐sectional	2013 to 2017	25,182	Addressing cost and time barriers in chronic disease management through telemedicine: An exploratory research in select low‐and middle‐income countries	*Therapeutic Advances in Chronic Disease*
Sclafani et al. (2021)	US	Survey	March through August 2020	NR	Telemedicine lessons learned during the COVID‐19 pandemic: The augmented outpatient otolaryngology teleconsultation	*American Journal of Otolaryngology–Head and Neck Medicine and Surgery*
Singh et al. (2022)	US	Descriptive study	NR	NR	Telemedicine during COVID‐19 crisis and in post‐pandemic/post‐vaccine world‐historical overview, current utilization, and innovative practices to increase utilization	*Healthcare*
Wootton et al. (2005)	Australia	Descriptive study	NR	33	E‐health and the universities 21 organization: 2. Telemedicine and underserved populations	*Journal of Telemedicine and Telecare*
Zahid et al. (2022)	Pakistan	Cross‐sectional	April to June 2022	100	Perception and attitude of Pakistani doctors toward the use of telemedicine technology	*Cureus*

a NR: Not reported.

### 3.1 Results of Syntheses

Based on the combination of the results of individual studies and the SWOT framework, the results are categorized into four domains: strengths, weaknesses, opportunities, and threats. Strengths included ease of use of technology, reduction of time and cost, and facilitating documentation. Weaknesses included a lack of physical exams, less direct communication, and internet‐related problems. The opportunities included the increasing progress of medical technologies worldwide, continuity of care, and people's interest in the daily use of new technologies. Threats included sociocultural barriers, the need for continuous training, and competing interests (Table 3).

**Table 3:** SWOT analysis of teleconsultation extracted from articles.

Domain	Core category	Subcategory	Number of articles (*n* = 32)	Exemplar quotes
Strengths	Reduction of time and cost	– Saving time due to no need to visit in person	25	Patients saved an average of 2 h per consultation by avoiding travel.
		– Saving money due to no need to visit in person	20	Transportation cost savings were reported by 78% of low‐income participants.
	Ease of use of technology	– User‐friendliness of technologies	18	The platform's intuitive design allowed even elderly patients to navigate it easily.
		– No need for complex skills to use technology	15	Most GPs required less than 30 min of training to use the system effectively.
	Facilitating documentation	– Facilitating documentation using the cloud	14	Cloud‐based records reduced administrative workload by 40%.
		– Saving information in the system	10	—
		– Ability to save information automatically	7	—
Weaknesses	Lack of physical exams	– Lack of proper diagnosis due to lack of physical examination	28	Misdiagnosis rates for dermatological conditions increased by 15% in virtual consultations.
		– Incorrect prescription due to lack of physical examination	13	—
	Less direct communication	– Inappropriate understanding of some service provider explanations	12	Patients often misunderstood dosage instructions without face‐to‐face clarification.
		– Less understanding of the caregiver's feelings and emotions by the service provider	9	—
	Internet‐related problems	– Internet interruption	26	35% of rural consultations were disrupted due to connectivity issues.
		– Low internet speed	8	—
		– High cost in some countries	7	—
		– Lack of proper infrastructure to provide services	12	—
Opportunities	Increasing progress of medical technologies in the world	– Increasing the growth of medical technologies	19	AI‐driven diagnostic tools are expected to enhance teleconsultation accuracy.
		– Increasing centers providing remote medical services	11	—
	Continuity of care	– The possibility of continuous follow‐up	17	Automated reminders improved medication adherence by 30%.
		– Sending an alarm to the recipient of the service to continue the treatment process	19	—
	People's interest in the daily use of new technologies	– Increasing people's use of technology continuously	20	—
		– Increasing the use of technology in all aspects of daily life	18	70% of patients under 50 preferred telehealth for routine care.
Threaten	Sociocultural barriers	– Cultural barriers: Preference for in‐person care due to distrust in technology (e.g., “Older adults feared technology would depersonalize care”)	12	In our community, seeing a doctor in person is a tradition; screens feel impersonal.
		– Moral barriers: Privacy concerns (e.g., “Patients worried about data leaks”)	10	Young women avoided discussing sensitive issues online due to privacy fears.
		– Religious and belief barriers (e.g., “Teleconsultation conflicts with spiritual healing practices”)	7	Some patients believe healing requires physical touch, which technology cannot provide.
	Need for continuous training	– Inadequacy of technology development with the training provided to the recipient and the service provider	16	GPs reported gaps in managing telehealth‐specific ethical dilemmas.
		– The need to update technology and thus update information	11	—
	Competing interests	– Conflict of interest on the part of the doctor	5	—
		– Conflict of interest on the part of the patient	4	—
		– Conflict of interest on the part of the service provider	11	—
		– Conflict of interest on the part of insurance companies	14	Insurers often denied claims for telehealth, citing insufficient evidence of efficacy.

## 4 Discussion

The use of telemedicine technologies in general and, teleconsultations in particular is increasing daily. On the other hand, these methods have good and useful features and various disadvantages. These methods should be chosen considering the conditions of the service recipient, the service provider, the context, and the type of service [55, 56, 57].

Due to the COVID‐19 pandemic, the use of teleconsultation has promoted many healthcare systems, and it has strengthened, the most important reasons for which were the lack of need to visit people and maintain social distancing to prevent this disease. On the other hand, due to the reduction in the cost and time required to receive care or service, the use of this method has increased in recent years. Another reason is the increase in all technologies used in daily life, which includes the use of remote medical technologies and teleconsultation. Due to the complexities of city life and the impossibility of visiting people in person, many people prefer to receive teleconsultation services [30, 36, 39, 40, 58].

Using SWOT analysis is a useful method for understanding the status of emerging technology. This tool helps us to identify the situation of technology in the industry; thus, we can choose the best strategies. These strategies can be selected by considering internal and external factors. The internal factors include strengths and weaknesses, and the external factors include opportunities and threats. Knowing these four domains about any technology can help policymakers start and use a technology considering the current situation [59, 60, 61].

The results of these studies indicate that the effectiveness of teleconsultation can reach the same level as that of face‐to‐face consultation. Of course, it should be taken into account that this method is not suitable for all patients, and clinical conditions, the severity of the disease, type of disease, patient's condition, and some side effects should be considered. This method is not recommended for patients who have acute conditions and need a physical examination. This method is suitable for patients who have movement or travel problems or patients who do not need a physical examination. This approach will also be useful for patients whose treatment needs continuous follow‐up. Usually, the use of video conferencing is more suitable for younger patients, and elderly people usually prefer to use the phone [56, 57, 62, 63, 64, 65, 66, 67].

Another discussion that can be very important is in terms of justice and equality. The use of teleconsultation can help increase equity and equality because it makes access easier for all groups of patients. This issue is especially useful for less privileged areas and rural areas where there are fewer facilities. On the other hand, examination time is usually shorter in teleconsultations, which can save people time and cost. Some evidence suggests that teleconsultation can decrease the cost of service and the cost per episode [63, 68].

It seems that expanding the use of teleconsultation can lead to better patient integration of information and communication. However, there are some concerns regarding the existence of conflicts of interest between individuals and the need for continuous training of service providers in this field. Usually, people who have a better relationship with their doctor are more inclined to use teleconsultation, and this doctor‒patient relationship is well understood [23, 28, 38, 53, 62, 65].

A study titled “Analysis of strengths, weaknesses, opportunities, and threats in the field of telemedicine” was conducted in Egypt. In this study, researchers identified four trends in telemedicine use for 2020 from governmental, financial, technical, and medical perspectives. The main finding of this study is that telemedicine will be a part of the national electronic health plan. However, it has not yet been used. One of the reasons for this is resistance to change, the lack of a business model for telemedicine, and the lack of proper infrastructure for this technology [26].

The results of Ehtashami et al.'s study, which was conducted to develop telemedicine strategies at Isfahan University of Medical Sciences, showed that most of the strengths are related to educational and research factors, and the weakest are related to legal considerations, equipment, and standards. Most of the shortages are related to human resources. The greatest relative importance of opportunities was related to “the existence of international telemedicine experiences and case studies,” and the greatest relative importance of threats was related to “telemedicine innovation in Iran and lack of sufficient experience for part of the university [69].”

## 5 Conclusion

According to the studies conducted, a set of factors can be effective in ensuring the success and failure of telemedicine projects. In general, according to the findings of the present research, the results indicate that, under the current conditions, not only will these cases be considered, but also the adoption of appropriate plans to minimize weak points and threats and the effective use of strengths and opportunities to implement projects in the field of telemedicine will probably fail, wasting time, effort, and funds in the health sector. Therefore, it can be concluded that with proper planning and the use of strengths and opportunities to eliminate weaknesses and threats in the future, we can witness the provision of health services with the help of information and communication technologies and take advantage of its advantages in increasing access to services and ultimately increasing justice and equality in the provision of health services.

## 6 Study Limitations

This systematic review was limited to articles in the English and Persian languages. Adding the results of other studies in other languages may have caused differences in our results. Of course, in general, the studies are aligned, and the changes will be minor. Another limitation of this study is that the use of teleconsultation was investigated in general and not in specific patient groups or different age groups; these topics will be investigated by researchers in the future. The focus here has been more on the feasibility of using teleconsultation.

## Author Contributions

Shiva Khoshsirat collected data. All authors analyzed and interpreted the data and prepared the first manuscript draft. All authors contributed significantly and critically to the final manuscript. All authors read and approved the final manuscript. All authors have read and approved the final version of the manuscript. The corresponding author had full access to all of the data in this study and takes complete responsibility for the integrity of the data and the accuracy of the data analysis.

## Ethics Statement

The authors have nothing to report.

## Consent

The authors have nothing to report.

## Conflicts of Interest

The authors declare no conflicts of interest.

## Transparency Statement

The lead author, Peyman Rezaei‐Hachesu, affirms that this manuscript is an honest, accurate, and transparent account of the study being reported, that no important aspects of the study have been omitted, and that any discrepancies from the study as planned (and if relevant, registered) have been explained.

## Data Availability

The data that support the findings of this study are available from the corresponding author upon reasonable request.
